# Mechanic’s hands revisited: is this sign still useful for diagnosis in patients with lung involvement of collagen vascular diseases?

**DOI:** 10.1186/1756-0500-7-303

**Published:** 2014-05-17

**Authors:** Erei Sohara, Takeshi Saraya, Shinji Sato, Naoki Tsujimoto, Takayasu Watanabe, Saori Takata, Yasutaka Tanaka, Haruyuki Ishii, Hajime Takizawa, Hajime Goto

**Affiliations:** 1 Department of Respiratory Medicine, Kyorin University School of Medicine, 6-20-2 Shinkawa, Mitaka City, Tokyo 181-8611, Japan; 2 Division of Rheumatology, Department of Internal Medicine, Tokai University School of Medicine, Tokai University School of Medicine, 142 Shimokasuya, Isehara 160-8582, Japan

**Keywords:** Anti-aminoacyl-transfer RNA synthetase (ARS) syndrome, Dermatomyositis, Mechanic’s hands, Skin lesions

## Abstract

**Background:**

The presence of “mechanic’s hands” is one of the clinical clues for collagen vascular diseases. However, the exact relevance of “mechanic’s hands” in collagen vascular diseases has not been well documented. The aim of this study was to clarify the relevance of “mechanic’s hands” to collagen vascular diseases including various skin lesions and interstitial pneumonia.

**Methods:**

A retrospective review of the medical records of patients with “mechanic’s hands” at our hospital between April 2011 and December 2012 was conducted. A PubMed search was also conducted using the term “mechanic’s hands”.

**Results:**

Four patients in our institution and 40 patients obtained from PubMed who had “mechanic’s hands” were identified. The most frequent diseases were DM/amyopathic DM (n = 24, 54.5%) and anti-ARS syndrome (n = 17, 38.6%). In these patients, the major skin lesions associated with “mechanic’s hands” were periungual erythema (n = 23, 52.3%), Gottron’s sign (n = 17, 38.6%), heliotrope rash (n = 10, 22.7%), Raynaud’s phenomenon (n = 9, 20.5%), and anti-ARS syndrome (n = 17, 38.6%). Six cases (2 DM, 4 anti-ARS syndrome) had only “mechanic’s hands”. Antibodies to anti-ARS (n = 24) were Jo-1 (n = 19), PL-7 (n = 3), OJ (n = 1), and PL-12 (n = 1).

**Conclusion:**

The presence of “mechanic’s hands” together with diverse skin lesions could be a clinical clue to the diagnosis of lung involvement associated with collagen vascular diseases, especially in anti-ARS syndrome or DM/amyopathic DM.

## Background

In 1979, Stahl et al. [1] described “mechanic’s hands” as a hyperkeratotic eruption on the ulnar aspect of the thumb and radial aspect of the index finger, with desquamation and rhagades. The presence of “mechanic’s hands” has been reported to be highly relevant in patients with collagen vascular-related interstitial pneumonia, dermatomyositis, systemic lupus erythematosus, and mixed connective tissue disease. In the modern era, anti-ARS syndrome has emerged as a new clinical entity associated with interstitial pneumonia. However, the exact relevance of “mechanic’s hands” in these various diseases has not been well reported. Four patients with amyopathic dermatomyositis or dermatomyositis with “mechanic’s hands” are reported, and 40 previously reported cases are reviewed.

## Methods

Patients who presented to the Kyorin University School of Medicine (Mitaka City, Tokyo, Japan) who were consecutively admitted to the Department of Respiratory Medicine with “mechanic’s hands” based on the criteria of Stahl et al. were investigated [1]. A retrospective study over a 20-month span from April 2011 was conducted. Patients who satisfied the criteria for inflammatory myositis, polymyositis, and dermatomyositis proposed by Bohan and Peter’s [2] and Tanimoto et al. [3], respectively, were enrolled. The medical literature was also searched using PubMed to identify reports of “mechanic’s hands”. This retrospective study was approved by the Ethics Board of Kyorin University.

## Results

**Figure 1:**
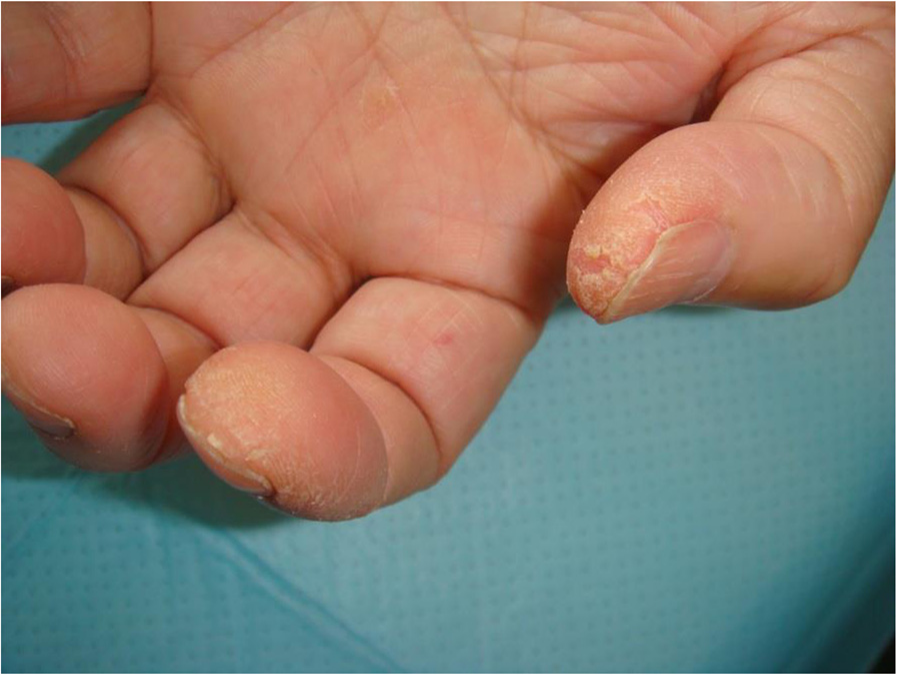
Patient 3 has fissures and roughness with hyperkeratosis and scaling on the pulp of the thumb and the radial aspect of the index finger.

In our institution, 10 consecutive patients with DM (n = 9) or DM/SLE overlap syndrome (n = 1) were identified, of whom four had “mechanic’s hands” (Figure 1 of case 2).

Case 1 was a 51-year-old man who presented to our emergency department with a 10-day history of dyspnea and dry cough. He had a medical history of atopic dermatitis and bronchial asthma. His vital signs were as follows: blood pressure 130/80 mmHg, pulse rate 96 beats/min, temperature 36.8°C, respiratory rate 24 breaths/min, and oxygen saturation 99% at 6 L/min oxygen delivered via a mask. Physical examination was normal except for inspiratory fine crackles in bilateral lower lung fields posteriorly. He had skin lesions of “mechanic’s hands” on the ulnar aspect of the thumb and radial aspect of the index finger, as well as erythema at the nose, anterior portion of the neck or back, and olecranon 1 month prior to coming to our hospital. The skin over the palmar and dorsal aspects of the finger joints of both hands showed red-purple, keratotic, atrophic erythema, suggesting Gottron’s sign, together with periungual erythema. Dark red erythema with hyperkeratosis was found on the anterior portion of the neck and back, compatible with the V-neck sign and shawl sign, respectively.

Laboratory data revealed slightly elevated levels of C-reactive protein (CRP) (2.8 mg/dL) and aldolase (22.6 U/L). Moderate elevations of serum lactate dehydrogenase (LDH (413 IU/L), KL-6 (883 U/mL), and SP-D (222 ng/mL) were also noted. Arterial blood gas analysis with oxygen at 6 L/min via mask showed hypoxemia (80.9 Torr), but other data were normal (pH 7.444, pCO_2_ 37.8 Torr, and HCO_3_- 24.1 mEq/L). Antibody to clinical amyopathic dermatomyositis (CADM)-140 autoantigen antibody (titer 1:232) was detected, but no anti-aminoacyl-transfer RNA synthetase (anti-ARS) antibodies including Jo-1 were detected. Thoracic CT showed an organizing pneumonia (OP) pattern.

He satisfied five of the nine Japan College of Rheumatology criteria [3], and was thus diagnosed with amyopathic dermatomyositis (DM) with interstitial pneumonia (IP). He was immediately treated with intravenous steroid pulse therapy (methylprednisolone 1 g/day for 3 days), followed by oral maintenance steroid therapy with a dose of 0.8 mg/kg/day combined with oral cyclosporine (4 mg/kg/day). A few days after initiation of treatment, his skin lesions and radiological abnormalities disappeared almost completely, while his respiratory failure improved gradually within a month, and he was discharged uneventfully.

Case 2 was a 71-year-old male artist (painter) who presented to our hospital due to exertional dyspnea and low-grade fever for a month. His medical history included a thyroid tumor and prostate cancer at 26 and 62 years old, respectively. An abnormality was noted on a chest radiograph 10 months prior to coming to our hospital, but he denied any diagnostic procedures. His vital signs were as follows: blood pressure 120/78 mmHg, pulse rate 60 beats/min, temperature 36.6°C, respiratory rate 20 breaths/min, and oxygen saturation 94% on ambient air. Physical examination was normal except for inspiratory fine crackles in posterior lower lung fields bilaterally. He had “mechanic’s hands”, Gottron’s sign, and periungual erythema.

Laboratory examination showed mild elevation of LDH (325 UI/L) and moderate elevation of SP-D (223 ng/mL). KL-6 was markedly elevated (1,817 U/mL). Anti-Jo-1 antibody was detected (titer 1:174.9), but no other auto-antibodies, including anti-ARS antibodies, were detected. Thoracic CT showed curvilinear opacities in the right lower lobe, suggesting non-specific interstitial pneumonia (NSIP) pattern. He was clinically diagnosed with amyopathic DM with IP and was treated with oral prednisolone 0.8 mg/kg/day. His skin lesions and radiological abnormalities improved gradually, and he was successfully discharged on hospital day 30.

Case 3 was a 31-year-old man who presented to our hospital with a 2- month history of hyperkeratotic papules on bilateral fingers and erythema on the nose. He had a low-grade fever, arthralgia, myalgia, and muscle weakness in the upper extremity for 1 week. His vital signs were normal except for low-grade fever (37.2°C). He had “mechanic’s hands” on the ulnar aspect of the thumb and radial aspect of the index finger and Gottron’s sign or periungual erythema of bilateral fingers.

Laboratory examinations were normal except for slightly elevated levels of LDH (316 IU/L), while the presence of antibody to CADM-140 autoantigen (titer 1; 231) was noted, but no other auto-antibodies were detected. Thoracic CT showed patchy consolidation with bronchiectasis at the bottom of the lung, predominantly in the right thorax, suggesting NSIP pattern. He satisfied five of the nine Japan College of Rheumatology criteria [3], and biopsy of the erythema of his left index finger showed superficial perivascular and interface dermatitis, which was histologically identical to that of DM. He was thus diagnosed with amyopathic DM with IP, and discharged on hospital day 3 without treatment. He was in good health for the next 5 months without treatment, and most skin lesions and abnormal findings on thoracic CT disappeared spontaneously in this period, but 6 months after diagnosis, he returned to our hospital with abrupt onset of photophobia, dysphagia, aphasia, and numbness of the left sided-trunk, suggesting a central nervous system disorder. T2-weighted magnetic resonance imaging (MRI) demonstrated hyperintense lesions at the periventricular white matter that extended longitudinally in the spinal cord from C5 to Th8 (figure not shown). The cerebrospinal fluid showed pleocytosis (total cell count 2 cells/μL, total protein 46.9 mg/dL), and serum anti-aquaporin-4 (AQP-4) antibody was positive. He was finally diagnosed with amyopathic DM accompanied by neuromyelitis optica (NMO). He was then treated intensively with four cycles of steroid pulse therapy (prednisolone 1 g/day for 3 days), followed by maintenance therapy with oral prednisolone (0.5 mg/kg/day), and discharged uneventfully.

Case 4 was a 77-year-old man who presented to his local hospital due to erythema of the face and body. The erythema progressed, and muscle weakness in the thigh, as well as eyelid edema, emerged. Two months after his first visit to his local hospital, he came to our hospital. His vital and physical examinations were normal except for cutaneous lesions such as “mechanic’s hands”, Gottron’s sign, periungual erythema, heliotrope rash, shawl sign, and V-neck sign. Laboratory examinations showed slightly elevated white blood cells (WBCs) (9100/μL) and LDH (311 IU/L), and marked elevation of CK (509 IU/L). Antinuclear antibody was detected (titer 1:640), but no other auto-antibodies were detected. He satisfied four of the nine Japan College of Rheumatology criteria [3]. He had no lung involvement and was thus diagnosed with DM. At the same time, he was diagnosed with gastric cancer, which was treated by total gastrectomy. After surgery, he suffered from exacerbation of myositis, but intravenous steroid pulse therapy (prednisolone 1 g/day for 3 days), followed by intravenous maintenance therapy with prednisolone (1 mg/kg/day), led to complete resolution of the myositis, and he was transferred to another hospital.

Along with an intensive review of the previous literature, 44 patients with “mechanic’s hands”, including the four present cases, were identified. Their age was 51.5 ± 16.3 (mean ± S.D) years and ranged from 7 to 79 years, with a male to female ratio of 18:26 (Table 1) [4-25]. They had diverse diseases, such as DM/amyopathic DM (n = 24, 54.5%), anti-ARS syndrome (n = 17, 38.6%), sclerodermatomyositis (n = 2, 4.5%), and rheumatoid arthritis (n = 1, 2.3%) (Table 2). Skin lesions other than “mechanic’s hands” were noted, such as periungual erythema (n = 23, 52.3%), Gottron’s sign (n = 17, 38.6%), heliotrope rash (n = 10, 22.7%), the V-neck sign (n = 5, 11.4%), and the shawl sign (n = 4, 9.1%) (Table 3). Importantly, six patients (two DM, four anti-ARS syndrome) had “mechanic’s hands” as the sole skin lesion. The other 38 patients had multiple skin lesions with the following number of lesions other than “mechanic’s hands”: one (20 patients), two (11 patients), three (four patients), four (one patient), and five (two patients). The combinations of skin lesions are summarized in Table 4. The most common combination pattern of multiple skin lesions was periungual erythema and Raynaud’s phenomenon. On the other hand, two or more skin lesions with “mechanic’s hands” tend to be recognized as a combination of Gottron’s sign/periungual erythema and heliotrope rash/periungual erythema, in this order (Table 4). Interestingly, the proportion of patients with IP who had one (n = 17), two (n = 10), and three (n = 4) skin lesions in addition to “mechanic’s hands” was high, 85%, 91%, and 100%, respectively. This fact suggested that the presence of diverse skin lesions together with “mechanic’s hands” raises the possibility of lung involvement associated with collagen vascular diseases.

**Table 1:** Clinical features of patients with mechanic’s hands reported previously and the present four patients

**Year**	**Age (y)**	**Sex**	**Gottron’s sign**	**Heliotrope rash**	**Shawl sign**	**V-neck sign**	**Raynaud’s**	**Periungual erythema**	**Myalgia or weakness**	**Auto-antibody**	**Diagnosis**	**IP**	**Ref**
1994	69	F	-	-	-	-	-	-	+	Jo-1	DM	+	[2]
1996	7	M	+	-	-	-	+	+	+	ANA, ds-DNA, RF	SDM	N.A	[3]
2000	67	M	+	+	+	+	-	+	-	-	DM	+	[4]
2001	42	M	+	-	-	-	-	-	+	ANA	DM	-	[5]
2002	41	M	-	+	-	-	+	-	+	Jo-1	Anti-ARS synd	+	[6]
2002	40	F	-	+	-	-	-	-	+	Jo-1	Anti-ARS synd	+	[6]
2002	30	M	-	+	-	-	-	-	-	Jo-1	Anti-ARS synd	+	[6]
2003	48	F	-	-	-	-	-	+	-	RNP	RA	+	[7]
2003	55	M	+	+	-	-	-	-	-	Jo-1, RNP	DM	+	[7]
2003	29	F	-	-	-	-	-	-	-	Jo-1	DM	+	[7]
2004	56	F	+	-	-	-	-	-	-	PM-SCL	SDM	+	[8]
2005	62	F	+	-	-	-	-	-	-	PL-7	DM	+	[9]
2005	59	F	-	-	-	-	+	-	+	Jo-1	Anti-ARS synd	+	[10]
2006	30	M	+	+	-	-	+	-	-	PL-7	Anti-ARS synd	+	[11]
2007	41	M	+	-	-	+	-	-	-	Jo-1	Anti-ARS synd	+	[12]
2007	37	F	-	-	-	-	+	-	+	Jo-1	Anti-ARS synd	+	[13]
2007	73	F	-	-	-	-	+	-	-	Jo-1	Anti-ARS synd	+	[13]
2007	49	F	-	-	-	+	-	-	-	Jo-1	Anti-ARS synd	+	[13]
2008	56	F	-	-	-	-	-	-	-	ANA, Jo-1, SS-A	Anti-ARS synd	+	[14]
2009	56	M	+	-	-	-	-	+	-	PL-7	DM	+	[15]
2009	64	F	+	-	+	-	-	+	+	-	DM	+	[15]
2009	44	F	+	-	-	-	-	+	-	ANA	DM	-	[15]
2009	56	M	-	+	-	-	-	+	+	ANA	DM	+	[15]
2009	65	F	-	+	-	-	-	+	+	ANA	DM	+	[15]
2010	54	F	-	-	-	-	-	-	-	OJ	Anti-ARS synd	+	[16]
2010	67	F	-	-	-	-	-	+	-	SS-A, Jo-1	Anti-ARS synd	+	[17]
2010	48	F	+	-	-	-	+	+	-	SS-A, Jo-1, ASMA	Anti-ARS synd	+	[18]
2011	64	F	-	-	-	-	+	-	+	Jo-1	Anti-ARS synd	+	[19]
2011	43	M	-	-	-	-	-	-	+	Jo-1	Anti-ARS synd	+	[20]
2012	21	M	-	-	-	-	+	-	+	PL-12	Anti-ARS synd	+	[21]
2012	79	M	-	-	-	-	-	-	+	Jo-1	Anti-ARS synd	+	[22]
Case 1	51	M	+	-	+	+	-	+	+	CADM-140	ADM	+	
Case 2	71	M	+	-	-	-	-	+	-	Jo-1	ADM	+	
Case 3	31	M	+	-	-	-	-	+	+	CADM-140, AQP-4	DM	+	
Case 4	77	M	+	+	+	+	-	+	+	ANA	DM	-	

ANA: anti nuclear antibody, AQP-4: aquaporin 4, ARS: aminoacyl-transfer RNA synthetase, ASMA: anti-smooth muscle antibody, CADM: clinical amyopathic dermatomyositis, DM: dermatomyositis, ds-DNA: double strand-DNA, IP: interstitial pneumonia, NA: not available, NSIP: non specific interstitial pneumonia, OP: organizing pneumonia, PM: polymyositis, RA: rheumatoid arthritis, RF: rheumatoid factor, RNP: ribonucleoprotein, SDM: sclerodermatomyositis, UIP: usual interstitial pneumonia.

**Table 2:** Diseases related to mechanic’s hands

**Disease**	**n (%)**
DM/amyopathic DM	24 (54.5)
Anti-ARS syndrome	17 (38.6)
SDM	2 (4.5)
RA	1 (2.3)

ARS: aminoacyl-transfer RNA synthetase, DM: dermatomyositis, SDM: sclerodermatomyositis, PM: polymyositis, RA: rheumatoid arthritis.

**Table 3:** Skin lesions with mechanic’s hands

**Skin lesion**	**n**	**(%)**
Periungual erythema	23	(52.3)
Gottron’s sign	17	(38.6)
Heliotrope rash	10	(22.7)
V-neck sign	5	(11.4)
Shawl sign	4	(9.1)
None	6	(13.7)

**Table 4:** Combinations of skin lesions excluding mechanic’s hands

**One**	**n**
Periungual erythema	9
Raynaud's phenomenon	5
Gottron’s sign	3
Heliotrope rash	2
V-neck sign	1
**Two**	
Gottron’s sign/Periungual erythema	5
Heliotrope rash/Periungual erythema	3
Gottron’s sign/Heliotrope rash	1
Gottron’s sign/V-neck sign	1
Heliotrope rash/Raynaud's phenomenon	1
**Three**	
Gottron’s sign/Raynaud's phenomenon/Periungual erythema	2
Gottron’s sign/Raynaud's phenomenon/Heliotrope rash	1
Gottron’s sign/Shawl sign/Periungual erythema	1

As for the auto-antibodies, 24 had anti-ARS (n = 24), which consisted of Jo-1 (n = 19), PL-7 (n = 3), OJ (n = 1), or PL-12 (n = 1), followed by anti-nuclear antibody (n = 14), SS-A (n = 3), and anti-RNP antibody (n = 2). Interstitial pneumonia and myositis were common, seen in 38 (86.4%) and 24 patients (54.5%), respectively. Anti-ARS syndrome (all 17 patients) and DM (24 of 26 patients, including amyopathic DM or SDM) patients had a higher morbidity rate from interstitial pneumonia (100% and 79.2%, respectively).

## Discussion

In the past two decades, the anti-ARS syndrome has been defined as the presence of one of the eight antisynthetase autoantibodies (anti-Jo-1, anti-PL-7, anti-PL-12, anti-EJ, anti-OJ, anti-KS, anti-Zo, anti-Ha) [23,26], but, except for Jo-1 antibody, they are detectable only in special laboratories. Anti-ARS syndrome has at least one of the following three clinical features: interstitial lung disease, inflammatory myopathy, or inflammatory polyarthritis [23]. However, previous reports noted that anti-ARS syndrome does not always show evidence of myositis, and a small percentage (2-11%) of patients with dermatomyositis does not have muscle involvement (so-called amyopathic dermatomyositis or dermatomyositis sine myositis) [14]. Thus, more attention should be paid to skin lesions in the diagnostic process. In our institution, there were no patients with anti-ARS syndrome during the study period.

“Mechanic’s hands” were first described by Stahl et al. in 1979 [1], with similar findings to manual workers, clinically resembling hand eczema [19]; however, the following differential points were noted: 1) there was no evidence of pruritus and vesicles; 2) the lesions were usually symmetrical, not related to the dominant hand; 3) involved the ulnar aspect of the thumb and the radial aspect of the fingers, most prominently on the index and middle fingers; and 4) liquefaction degeneration and colloid bodies were typical pathological features of “mechanic’s hands”. Previous reports showed that only 33% of patients with DM [1,27], as well as 30% to 70% of patients with anti-ARS syndrome [8], had “mechanic’s hands”. In this regard, in our institution, 40% of patients with DM had “mechanic’s hands”, a prevalence similar to that of previous reports. The present study demonstrated that the majority of patients with “mechanic’s hands” had anti-ARS syndrome or DM/amyopathic DM. However, this study had some limitations because it was a retrospective study, and one case series described by Sato et al. [25] was included in the present study that had a large number of patients (n = 9) with DM having “mechanic’s hands”. However, since the advent of the new clinical entity of anti-ARS syndrome in the last two decades, no study has been published describing the clinical significance of “mechanic’s hands”. In this regard, the present study showed that anti-ARS syndrome should be considered a leading cause of “mechanic’s hands”. Of note, six cases of patients with DM or amyopathic DM had “mechanic’s hands” as the sole skin lesion. Furthermore, only half of the patients in the present study showed myositis. Taken together, these facts suggest that the presence of “mechanic’s hands” serves as a high-yield guide in the diagnosis for DM/amyopathic DM or anti-ARS syndrome, which might be useful in cases that lack clinically apparent myositis and/or in institutions where detection of antisynthetase autoantibodies other than Jo-1 antibody is not available.

Although our study had some limitations in that all our patients had been admitted to the Department of Respiratory Medicine, lung involvement in dermatomyositis and idiopathic inflammatory myopathy occurs in 5-30% [10], while it is more common in anti-ARS syndrome (80%-100%) [24,27], as in the present study. Although no report has been published about “mechanic’s hands” associated with idiopathic interstitial pneumonia, Watanabe et al. demonstrated that antisynthetase antibody-positive cases accounted for 6.6% of idiopathic interstitial pneumonia cases [28]. From this perspective, when we encounter patients with IP, recognition of common combinations of diverse skin lesions, as well as “mechanic’s hands”, might be a clinical clue to the diagnosis of collagen vascular diseases associated with lung involvement.

## Conclusion

The presence of “mechanic’s hands” together with diverse skin lesions could be a clinical clue to the diagnosis of lung involvement with collagen vascular diseases, especially in anti-ARS syndrome or DM/amyopathic DM.

## Abbreviations

ARS: Anti-aminoacyl-transfer RNA synthetase syndrome.

## Competing interests

The authors declare that they have no competing interests.

## Authors’ contributions

ES and TS mainly wrote the manuscript. NT, TW, ST, YT, HI, HT, and HG managed the patients. SS examined the titer of anti-synthetase antibodies. All authors read and approved the final manuscript.
